# Macro-Scale Patterns in Upwelling/Downwelling Activity at North American West Coast

**DOI:** 10.1371/journal.pone.0166962

**Published:** 2016-11-28

**Authors:** Romeo Saldívar-Lucio, Emanuele Di Lorenzo, Miguel Nakamura, Héctor Villalobos, Daniel Lluch-Cota, Pablo Del Monte-Luna

**Affiliations:** 1 Instituto Politécnico Nacional, Centro Interdisciplinario de Ciencias Marinas (CICIMAR), La Paz, Baja California Sur, México; 2 School of Earth & Atmospheric Sciences, Georgia Institute of Technology, Atlanta, Georgia, United States of America; 3 Área de Probabilidad y Estadística, Centro de Investigación en Matemáticas AC, Guanajuato, México; 4 Centro de Investigaciones Biológicas del Noroeste, La Paz, Baja California Sur, México; University of California San Diego, UNITED STATES

## Abstract

The seasonal and interannual variability of vertical transport (upwelling/downwelling) has been relatively well studied, mainly for the California Current System, including low-frequency changes and latitudinal heterogeneity. The aim of this work was to identify potentially predictable patterns in upwelling/downwelling activity along the North American west coast and discuss their plausible mechanisms. To this purpose we applied the min/max Autocorrelation Factor technique and time series analysis. We found that spatial co-variation of seawater vertical movements present three dominant low-frequency signals in the range of 33, 19 and 11 years, resembling periodicities of: atmospheric circulation, nodal moon tides and solar activity. Those periodicities might be related to the variability of vertical transport through their influence on dominant wind patterns, the position/intensity of pressure centers and the strength of atmospheric circulation cells (wind stress). The low-frequency signals identified in upwelling/downwelling are coherent with temporal patterns previously reported at the study region: sea surface temperature along the Pacific coast of North America, catch fluctuations of anchovy *Engraulis mordax* and sardine *Sardinops sagax*, the Pacific Decadal Oscillation, changes in abundance and distribution of salmon populations, and variations in the position and intensity of the Aleutian low. Since the vertical transport is an oceanographic process with strong biological relevance, the recognition of their spatio-temporal patterns might allow for some reasonable forecasting capacity, potentially useful for marine resources management of the region.

## 1. Introduction

There are four coastal regions on the eastern edges of the main ocean basins that are characterized by low water temperatures and high concentrations of chlorophyll [1]: the California and Peru-Chile Currents in the Pacific Ocean and the Canary and Benguela Currents in the Atlantic Ocean [2]. These regions are also known to have socioeconomic importance because they contribute with approximately 20% of the global fishery catches and 5% of the total primary productivity in the oceans despite occupying less than 1% of the sea surface [3].

The elevated coastal upwelling activity that occurs in these four large current systems is the oceanographic event responsible for the remarkable biological productivity, which is unusually high relative to the rest of the ocean [2,4]. Among the upwelling systems which are associated to eastern boundary currents, the California Current (CC) is one of the most extensively studied and sampled portions of the ocean [5,6]. In the CC the wind-driven offshore/onshore transport determines the magnitude of seawater flow (Ekman transport), which is a spatially heterogeneous process [7] with episodically varying upwelling/downwelling activity, from weekly to multi-decadal and longer time-scales [8–10]. In this study we focused on analyzing the long term (decadal and multidecadal) and large scale (North American west coast) variability of the vertical water transport in the CC.

### 1.1. Study Area

The North Pacific Current is a broad eastward directed flow originated in the Kuroshio Extension current. When approaching the west coast of North America, the North Pacific Current (NPC) is divided into a subpolar branch that forms the Alaska Current (AC) and a subtropical branch that includes the California Current (CC). In the dominant mode of variability, the subpolar and subtropical gyres co-vary in response to fluctuations in the strength of the NPC [11], hence basin-scale dynamics play a major role in modulating the transport and strength of the AC and CC [12]. The Northwestern American coast comprises a large extension between latitudes 21 and 60°N. The upwelling/downwelling activity at this region is closely related to the physical state of the AC and CC.

The northern portion of the study area (~51–60°N) is under the domain of the Alaska Current (AC). The AC is a narrow, intense coastal current bordering the southern coast of Alaska. Relatively low salinities and some transport characteristics of the AC have been related to the regional regime of freshwater discharge [13]. Low pressure systems propagate east-northeast over this part of the North Pacific, occupying frequently the Gulf of Alaska and inducing significant changes on the prevailing hydrography [14]. The winds create downwelling conditions that reach maximum values in January and minima during the summer.

The California Current (CC) extends some 3000 km from the north of Vancouver Island (~42°N) to approximately the southern tip of the Baja California Peninsula (~23°N) [15]. The CC flows year-round with some seasonal patterns, reaches depths to about 500 m and is surface intensified by wind-stress [16]. Strong equatorward jets are embedded within the region of slower (~0.1 m∙s^-1^) southward flow [17]. The intense jets have widths of 50–75 km, speed in excess of 0.5 m∙s^-1^, and comprise up to half of the total CC transport. In some cases, these jets can be traced back to coastal upwelling jets that separate from the coast [16,18]. When wind-stress curl is negative, it strengthens the southward flow, resulting in a direct relationship between the seasonal and large-scale equator flow [7]. Equatorward winds onset summer offshore flow in the surface Ekman layer, leading to upwelling which typical velocities are 10–20 m∙d^-1^[6,7]

The coastal upwelling promoted by wind-stress in the CC is enhanced or attenuated by trapped coastal waves from local or remote origin [19]. Kelvin waves drive changes in the ocean density structure by means of the upwelling/downwelling activity propagated along the coast in direction to the pole at a speed of 30-90km∙day^-1^. Changes in oceanic density structure imply changes in the intensity of the current.

The southernmost station, at 21°N, shows hydrographic conditions than are different than those observed in other portions of the studied area. In winter and spring this region is mainly influenced by the CC, though mixed with waters from the Equatorial Countercurrent and the North Equatorial Countercurrent. In summer and autumn the CC weakens and the region is dominated by tropical waters from the North Equatorial Countercurrent [20,21].

### 1.2. Seasonal cycle

The winter starts with a gradual completion of summer conditions after the contraction of the North Pacific high pressure center, while the Aleutian low intensifies and moves to the east and south [22,23]. These changes result in the weakening of wind-driven upwelling, then the mixed layer thickens, the sea level decreases, the CC speeds up and advection of tropical water stops, leading to colder SST in the CC [6]. During the spring transition, a key seasonal event in the CC is forced by the winds blowing equatorward, generating southward transport along the coast and offshore Ekman transport [24].

The climatological summer in the CC consists in the relaxation of the Aleutian low pressure center and the intensification of the North Pacific high, which migrates westward and extends along most of the North Pacific basin [25]. In response, the atmospheric pressure gradient is diminished driving the weakening of winds and upwelling activity; the average speed of the CC slows down, sea level increases, thermocline and minimum oxygen layer deepen, the mixing layer is reduced, stratification in the water column increases, and both clorophyll-a and zooplankton concentrations decrease as consequences of the depleted primary productivity [23,26]. These changes in the physical environment and the increased advection of tropical waters cause the rise in sea surface temperature (SST) [6].

The changes in the amplitude and phase of seasonal upwelling events are linked to basin-scale seasonal dynamics through several mechanisms [27,28]. Some examples are the seasonal cycle evolution of the atmospheric pressure centers in the North Pacific (the Aleutian low and the North Pacific high) and their forcing on local winds that impact the upwelling/downwelling activity and, in consequence, also impact the ocean surface stratification and primary productivity [6,29,30].

However, the upwelling annual cycle is not a stationary process as it presents interannual variability on phenological properties (*e*.*g*. season onset and duration) that impact marine ecosystem productivity and determines the abundance of ecologically and economically important species [28,30]. For example; the late spring transition in upwelling season observed during the year 2005, impacted negatively to phytoplankton productivity and provoked large anomalies of biomass and composition of zooplankton [30].

### 1.3. Interannual patterns

Interannual fluctuations in ocean-atmosphere dynamics are determinant factors regarding the magnitude and direction of Ekman transport. The oceanic and atmospheric expressions of El Niño and the Southern Oscillation, correspondingly, are the main processes responsible of the year to year changes in the upwelling activity at the CC[6,31]: 1) in the ocean, trapped coastal waves propagate from the tropics towards high latitudes, deepening thermocline depth; and 2) in the atmosphere, teleconnections promote changes in the pressure gradients which results in weaker winds [31]. Both mechanisms result in a decreased vertical transport of nutrient-rich, cold water from the deep [31,32].

### 1.4. Low-frequency signals

After applying Singular Spectral Analysis (SSA) to upwelling time series from Northwestern American coast, [10] distinguished three energetic signals; semi-annual, annual and low-frequency. The annual signal accounted for 40% of total variability in the southern region of study area, and up to 87% around 33°N-36°N. The low-frequency signal resulted more important in the southern stations (>20%), but decreased progressively around 33°N. The latter signal (low-frequency) showed to be the second most important mode in upwelling variability.

Strong low-frequency signals are particularly useful regarding predictive models, which are increasingly used by decision makers at planning productive activities such as fishing or mariculture [22,33]. The present work is a contribution towards understanding the low frequency signals that modulate the intensity and direction of the Ekman transport. The aim of this work was to identify long term, large scale patterns of the upwelling activity along the North American west coast (21–60°N).

## 2. Methods

### 2.1. Data

**Fig 1:**
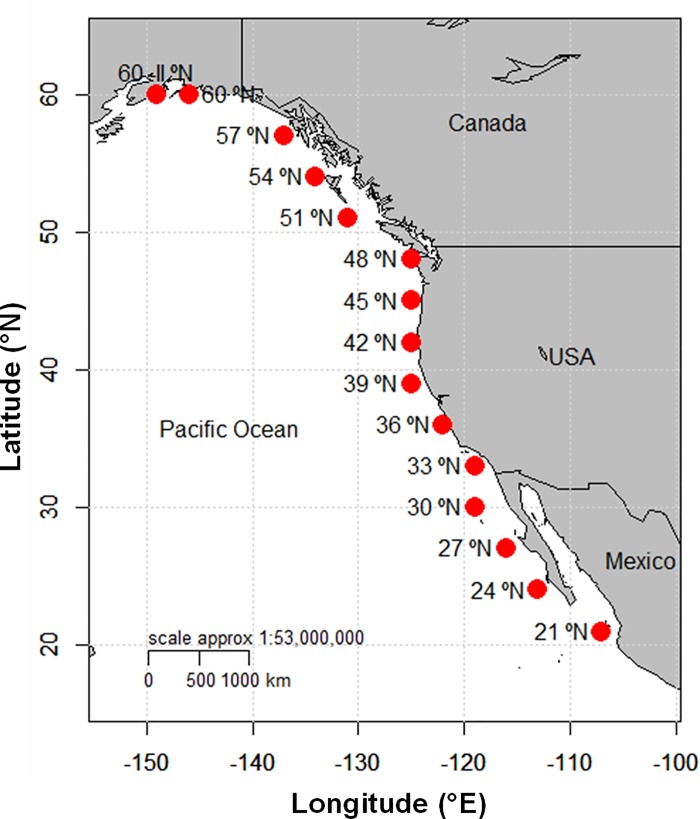
Stations where Upwelling Index is computed. Stations where the Fleet Numerical Meteorology and Oceanography Center (FNMOC) measures the atmospheric pressure fields, which are used by the Pacific Fisheries Environmental Laboratory (PFEL) to compute the upwelling index.

The Pacific and Fisheries Environment Laboratory (PFEL) developed an index that measures coastal upwelling intensities induced by winds in 15 locations along the coast of North America, between 21° and 60° latitude north (Fig 1). The index is based on estimates of Ekman transport caused by geostrophic wind stress. The geostrophic winds are derived from monthly averages of surface atmospheric pressure grid fields, which are recorded every 6 hours. This information is provided by the Fleet Numerical Meteorology and Oceanography Center of the United States Navy (FNMOC, Monterey, CA.). The idea behind this index was to develop a simple time series that can depict the long-term variation in coastal upwelling.

Monthly data corresponding to the upwelling indices recorded between 1946 and 2012 were downloaded from the PFEL web site: http://www.pfeg.noaa.gov/products/pfel/modeled/indices/upwelling/upwelling.html). Subsequently, the monthly values were added to obtain the total annual upwelled water by station.

Maximum Autocorrelation Factor Analysis (MAFA; see section 2.3.) was applied to upwelling index data in order to get a better signal-to-noise ratio, which facilitates the identification of periodicities within spectral analysis (section 2.4). The results of the spectral analysis were compared to those obtained by coupling cyclic descend and periodic regression analyses. Finally, skill assessment was conducted by including selected low-frequency signals in periodic regression models that were fitted to the first Factor of MAFA (MAF-1) and successive length-increasing time series were considered in a hindcast/nowcast approach.

### 2.2. Min/max Autocorrelation Factor Analysis (MAFA)

Originally developed to clean satellite signals emitted from multiple transmission channels [34], min/max autocorrelation factor analysis (MAFA) was adapted for the context of environmental and climatic variability [35]. Subsequently, other authors demonstrated the utility of this technique for analyzing common changes in fisheries and ecological data [36–38]. In this study, MAFA was an analysis tool that allowed exploring the spatial co-variation in macro scale upwelling/downwelling activity (21–60°N).

MAFA is based on linear transformations of the original series that meet certain characteristics for optimization. Unlike PCA (which seeks to successively maximize the variance of new variables built and projected on new orthogonal axes) MAFA seeks to maximize the serial autocorrelation of each new built series. Each output variable is a linear combination of all input series, and the autocorrelation criterion is what makes the technique particularly relevant to the context of time series. The primary objective of this procedure is that the new series, which are highly auto correlated, combine the information contained in the original series to enable the break-down and interpretation of their signal [35].

If the column vector of the *p* original series at time *t* is defined as

$$Z(t)=\left(Z_{1}\;(t),\ldots ,Z_{p}(t)\right){\;}^{T}$$ 1

The set of maximum autocorrelation factors (MAFs) obtained as outputs of the analysis (MAFA) are also *p* in number and can be denoted in a similar manner:

$$Y(t)=\left(Y_{1}\;(t),\ldots ,Y_{p}(t)\right){}^{T}$$ 2

Where *T* indicates the total number of the original time series and, therefore, the total number of MAFs. Then, MAFs are linear combinations of the originals, i.e.,

Y(t)=AZ(t) 3

where matrix *A* with dimensions *p* x *p* is fixed (is not time dependent). This matrix is calculated using linear algebra techniques and algorithms, ensuring that the following conditions are met:

1. The output series *Y*_1_(*t*),…,*Y*_*p*_(*t*), must be independent of each other.
2. The autocorrelation values with lag = 1 that correspond to the output series must be arranged in decreasing order.
3. Each series output must be positively correlated with time.

Because the autocorrelation with a lag of 1 is a measure of the degree of smoothness of a time series, these restrictions give *MAF* the following interpretation: the series *Z*(*t*) breaks down into a linear combination of orthogonal components of *Y*(*t*), where *Y*_1_(*t*) is the smoothest series possible, and the *Y*_*p*_(*t*) series is the least smooth of all the series. The total number of MAFs was determined just after all regions within the studied area were represented.

In addition to extracting the *MAF*_(n)_, canonical correlation analyses were performed [39] to determine the degree of association between the upwelling activity by station (original series) and the large-scale common variation (*MAF*_1…*p*_(*t*) = *Y*_1…*p*_(*t*)).

### 2.3. Spectral Analysis (SA)

Spectral analysis (SA) was applied to each normalized time series of the upwelling index as well as to the first six common signals (MAFs) identified by MAFA, which is explained in detail below. Firstly, the mean was extracted from the series and a tapering process was applied in order to minimize leakage using a 10% split-cosine-bell [40,41]. The Fast Fourier Transform was used to calculate the periodogram, which values were re-scaled to compensate the tapering effect in the order of 1/0.875. The spectral density was estimated with a Parzen spectral window of 5 terms.

Confidence bands of spectral densities were estimated from the Chi square distribution (D.F. = 10 and confidence bounds of 95, 90, 85 and 80%). The lower bounds of confidence intervals were compared to a “red-noise null spectrum” that was built with a larger window of Parzen smoother (~55 terms).

Though Fourier’s Transform considers a continuous time series, in practice spectral analysis is performed by sampling discrete time series, which implies analytic risks related to the contamination (“leakage”) of neighboring frequencies and potential mistakes at identifying a particular frequency (“aliasing”). Therefore, SA is regarded as an exploratory technique which does not guarantee an accurate identification of the “real” spectrum of frequencies present in the time series.

In order to evaluate the potential bias due to the aforementioned problems, SA was also applied to series of monthly (larger n) instead of annual, upwelling anomalies, with a 13 terms moving average filter. In order to strengthen the selection of the most important signals, a complementary criteria was applied by means of the combination of three different techniques: cyclic descend [42], periodic regression [43], and a standard F test [44] for identifying the number of significant periodic components on each time series, just as detailed by [45].

Once the most consistent periodicities were identified, they were included in periodic regression models to evaluate their relative contribution to nowcast/hindcast models regarding MAF-1. To achieve this, the length of time series considered at each fitting was increased gradually (*e*.*g*. by including the corresponding next observation as the new last value of the series). The estimated values were compared to the observed data (1946–2012).

Finally, we conducted a predictive skill assessment of regression models: we compared model fittings when using selected low frequency signals versus (false) signals from random numbers in a value range 1:100 and introducing equal quantity of signals (five). For this purpose, some statistics recommended by [46] were considered (Table 1). Statistical analysis were conducted with the support of statistical package [47].

**Table 1:** Skill assessment statistics applied to upwelling low frequency-based models.

SKILL STATISTIC	EXPLANATION
*R*	Correlation coefficient: observed *vs*. predicted
Positive Outlier Frequency (%)	Fraction (percentage) of Errors greater than the mean.
Negative Outlier Frequency (%)	Fraction (percentage) of Errors less than the mean.
Root Mean Square Error	$RMSE=\sqrt{\frac{1}{N}}\sum_{t=1}^{N}e_{i}^{2}$

## 3. Results

### 3.1. MAFA performance

**Fig 2:**
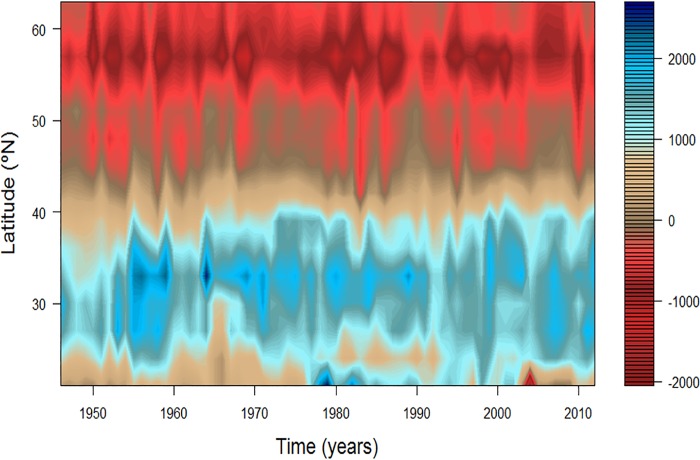
Total amount of upwelled/downwelled water per year (m^3^/s/100_m coastline_). Ekman transport on the west coast of North America between 21 and 60°N, according to the upwelling index (http://www.pfeg.noaa.gov/products/).

**Fig 3:**
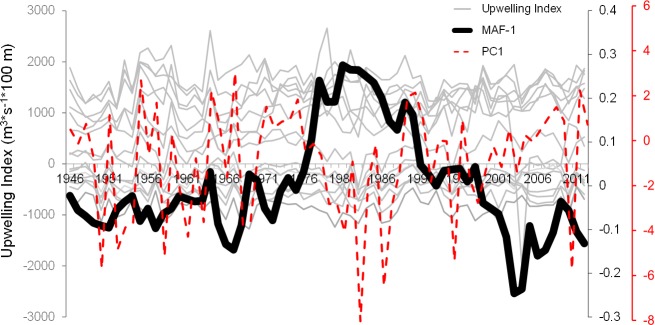
Upwelling/downwelling and the highest auto correlated signal, *MAF*-1. Upwelling indices from the 15th standard positions, 21–60°N (Fig 1; gray thin lines), and the extracted low frequency signal *MAF*-1 (black solid line). For comparison to *MAF*-1 it is shown the first principal component of upwelling (red dashed line).

**Fig 4:**
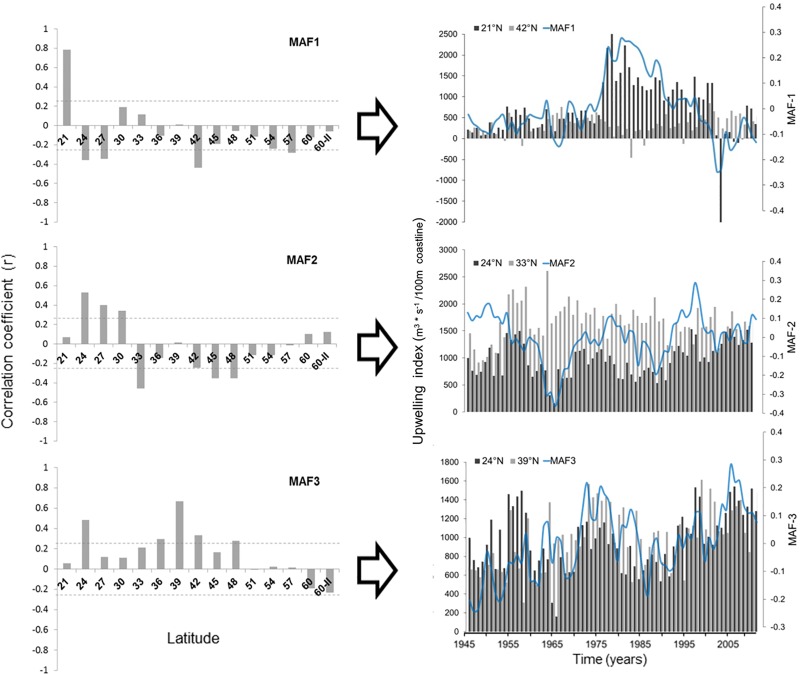
Canonical correlation coefficients between the original series (Ekman transport values by station) and extracted series (*MAFs* 1–3).

**Fig 5:**
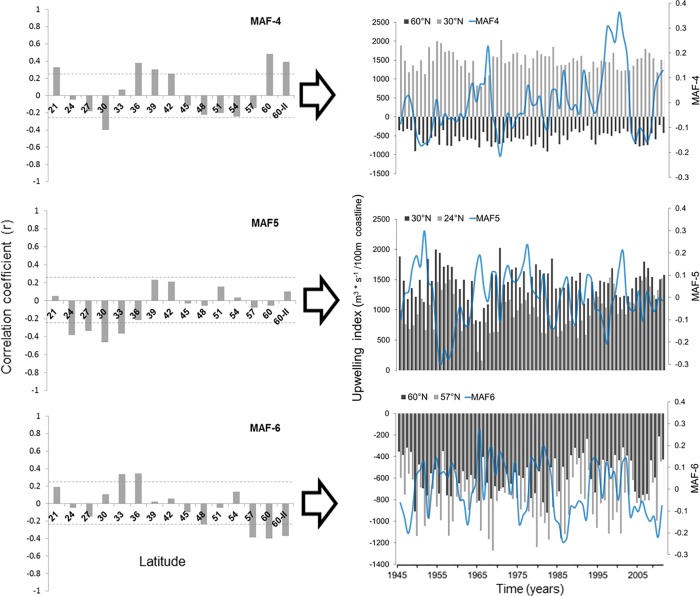
Canonical correlation coefficients between the original series (Ekman transport values by station) and extracted series *MAF* 4–6.

**Fig 6:**
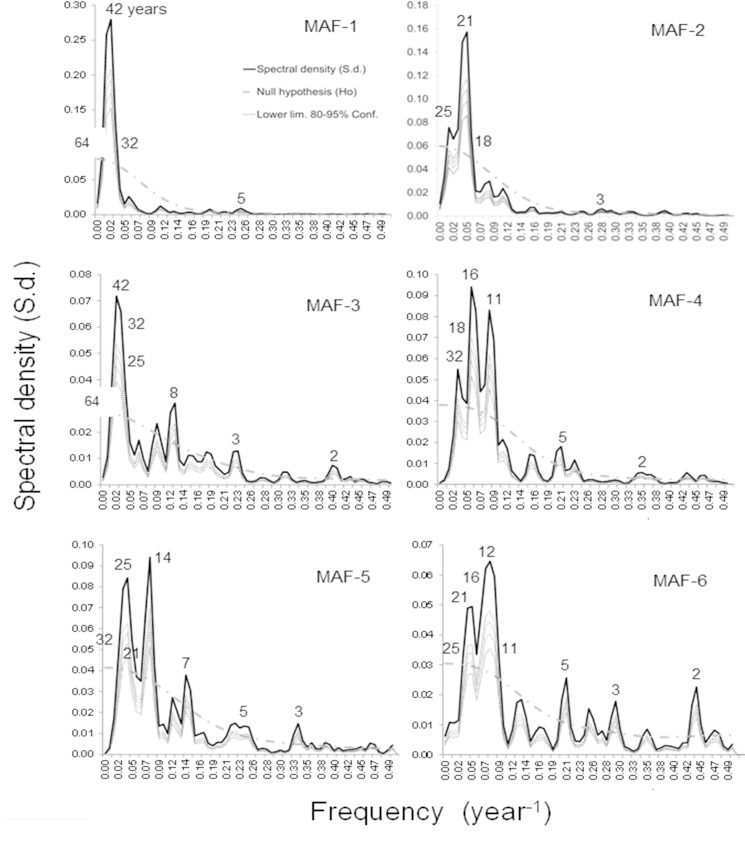
Spectral densities of common upwelling/downwelling signals (MAFs 1 to 6). Numbers indicate periodicities in years as reference values, which were statistically significant in a 80–95% confidence range.

**Fig 7:**
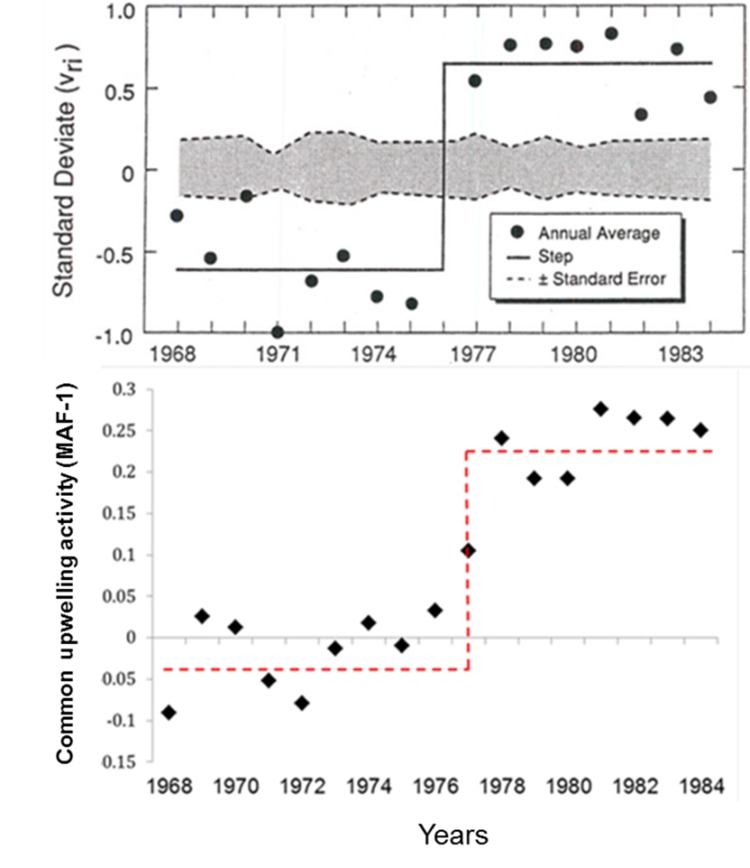
The 1976–77 regime shift and the *MAF*-1. Comparison of the North Pacific regime shift figure elaborated by Ebbesmeyer et al. (1991)[58] and the upwelling/downwelling change captured by the *MAF*-1. Original source of upper panel: PACLIM, 1991.

**Fig 8:**
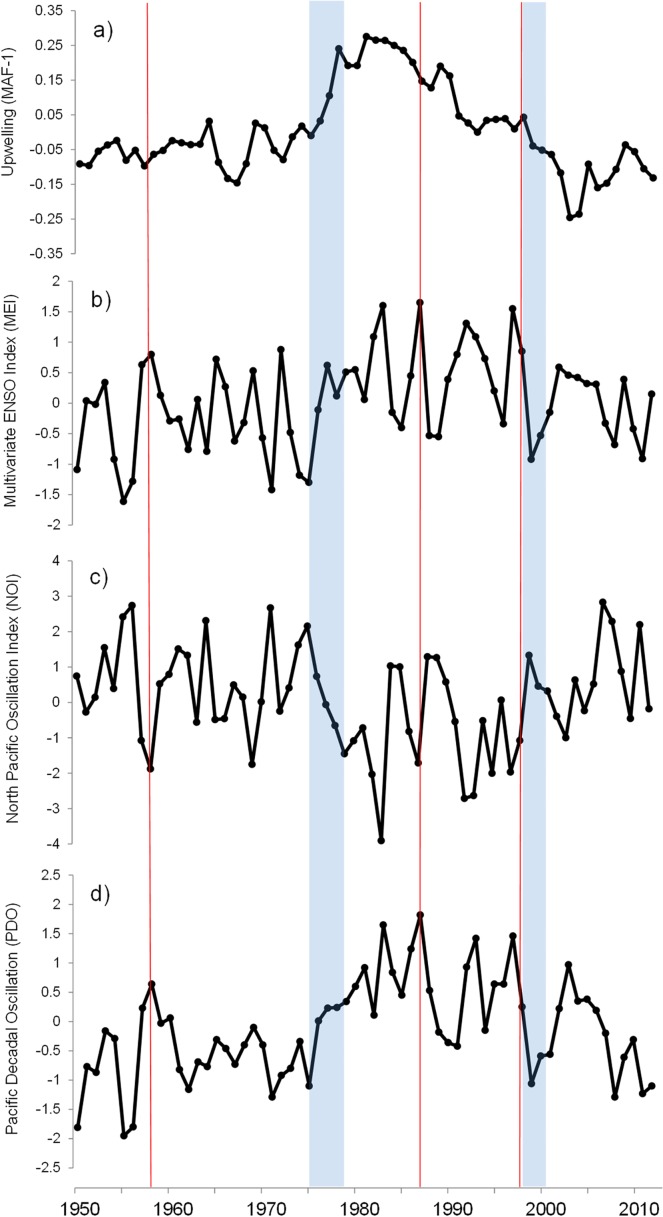
MAF-1 and climate indices. Time-series (1950–2012) comparison of the low-frequency signal in upwelling activity *MAF*-1 (upper panel), the Multivariate ENSO index MEI (b); the North Pacific Oscillation index NOI (c), and the Pacific Decadal Oscillation PDO (d). Blue shades indicate regime shifts widely accepted for the Eastern North Pacific. Thin red lines indicate three major El Niño events; 1958–59, 1987–88 and 1997–98.

Autocorrelations of the first six MAFs had values between 0.43 and 0.71 (p<0.001). The order of MAF(1…6) is indicative of autocorrelation level (smoothness). In other words, the first few MAF(1–3) contain lower frequency signals (Figs 2–6). The changes in each MAF(n) series, denote (spatially) simultaneous transitions that result from proportional or inverse intensification/reduction of Ekman transport among sampling locations (Figs 3–5). Thus, changes in the amplitude of Ekman´s transport were analyzed on the basis of strength and sign of correlations between each MAF(n) and the original upwelling index time series (per location; Figs 4 and 5). MAF-1, for example, had the strongest relationship with station 21°N (r = 0.78), followed by station 42°N (r>0.4; Fig 4). Between 1946 and 1976 MAF-1 presented relatively low values (Fig 3) corresponding to less than 750 m^3^/s/100_m coastline_ of annual flow in the southern station 21°N (Figs 2 and 4); the following years, annual flow increased to about 2700 m^3^/s/100_m coastline_ (peaking at 1979; Figs 2 and 3). The sudden increase in Ekman transport coincide with the regime shift (1977–79) widely recognized at Northeast Pacific region (Figs 7 and 8). Regime-like signal also was captured by MAF-1 which showed relatively high values since late 1970s until 1999 (Figs 3 and 7), corresponding to annual flows preferentially above 1000 m^3^/s/100_m coastline_ at 21°N (Fig 2).

The anti-correlation of station 42°N with MAF-1 (r = -0.44), and in consequence with station 21°N, was evident in the period 1976–2000, when upwelling trends were opposite between latitudes (Fig 4). At that time, 42°N station had values below 300 m^3^/s/100_m coastline,_ showing downwelling predominance in three different years (1983, 1986 and 1995) when Ekman transport was as negative as -457 m^3^/s/100_m coastline_ (Fig 2). The changes in the magnitude of transport had a range of ~1200 m^3^/s/100_m coastline_ (Figs 2 and 4). Stations 21°N and 42°N correlated positively during the period from 1946 to 1976, but no clear relationship was found after the 1999–2000 regime shift (Fig 4).

MAF-2 correlated positively with upwelling index at 24, 27 and 30°N: r = 0.53, 0.40 and 0.34, respectively (Fig 4). For stations at latitudes 33°N, 45°N and 48°N, statistically significant relationships with MAF-2 were negative (r = -0.46, -0.35 and -0.35). For 1998–99 years, the total amount of upwelled water was relatively high at 24°N, exceeding 1400 m^3^/s/100_m coastline._ This condition also persisted in 1955–1958 and 2005–2011; this three periods of high Ekman transport correspond to high values of MAF-2. In opposite way, high values of MAF-2 in 1946–58, 1971–88 and 1991–2003, correspond to a reduced Ekman transport at 33°N where changes in amplitude were about ~800 m^3^/s/100_m coastline_ (Fig 4).

Most stations correlated positively with the signal captured by MAF-3, with higher coefficients at 39°N (r = 0.67) and 24°N(r = 0.48). At both stations the upwelling activity had similar increasing/decreasing behavior, but upwelling values of particular years are out of phase (e.g. MAF-3 in Fig 4). For example, it can be observed a positive trend of MAF-3 during 1986–2007, changing amplitude about ~1000m^3^/s/100_m coastline_ at each station: in the northern station (39°N) the Ekman transport reached its highest value (1614m^3^/s/100_m coastline_) in 1999, meanwhile in the south (24°N) the peak value (1542 m^3^/s/100_m coastline_) was reached in 2007, eight years later (Fig 4). In complement, the minimum upwelling activity (550m^3^/s/100_m coastline_) started in 1990 in the south (24°N) and five years later in the north (39°N; MAF-3 in Fig 4).

The signal captured by MAF-4 presented more homogeneous relationships among single locations; relatively higher around 60°N (r = 0.49) and 30°N (-0.40), also being significate at the south, 21°N (r = 0.33), and center portions of the study area (36°N-42°N; r>0.31). Changes in MAF-4 related simultaneously to negative Ekman transport (downwelling) at 60°N and positive Ekman transport (upwelling) at 30°N (Fig 5). Otherwise, MAF-5 correlated significantly (r > -0.34) in southern stations, between 24°N and 33°N, with the highest coefficient at 30°N (r = -0.46). Fig 5 shows that positive changes in MAF-5 indicate a simultaneous depletion of Ekman transport at 24°N and 30°N, corresponding to variations about 700 m^3^/s/100_m_ and 600 m^3^/s/100_m_ in amplitude, respectively. A slight negative trend can be observed in MAF-5, apparently associated with differences in upwelling magnitudes between stations 24°N and 30°N in the last three decades; Ekman transport has increased at 24°N, while decreasing at 30°N (Fig 5).

Lower panel of Fig 5 shows the relative weight of upwelling/downwelling activity at 33°N (r = 0.34) and 36°N (r = 0.35) over MAF-6, with a heavier influence (r> -0.37) of northern stations (57 a 60°N; r> -0.37). The MAF-6 transitions indicate Ekman transport changes in the order of ±300 m^3^/s/100_m coastline_ at 60°N and ±400 m^3^/s/100_m coastline_ at 57°N. When MAF-6 values are high and positive, then downwelling intensifies at both latitudes, but if downwelling increases at one station while decreases in the other, then MAF-6 values turn negative (simultaneous change but in opposed directions; Fig 5).

### 3.2. Low-frequency signals

**Fig 9:**
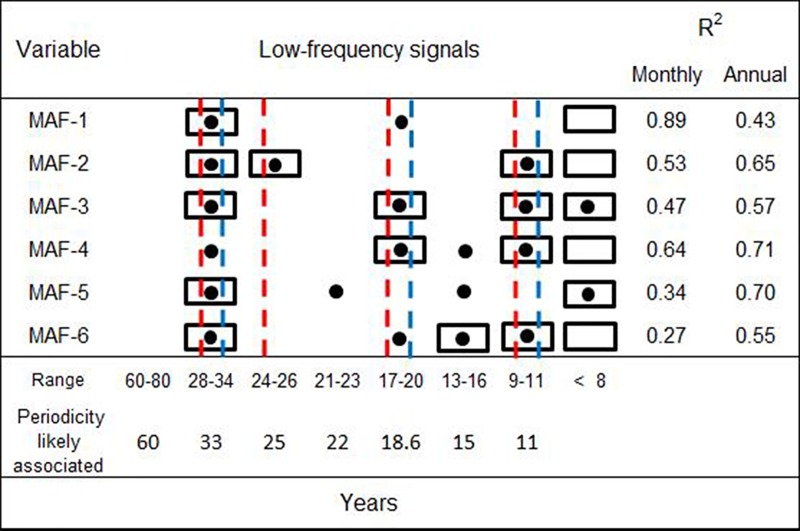
The vertical dashed lines show energetic spectral regions accordingly to Fast Fourier Transform for annual (red) and monthly (blue) resolutions of the low-frequency signals found in upwelling activity (MAFs). In addition, results from cyclic descend and periodic regressions for each MAF are shown in annual (black circles) and monthly resolutions (black rectangles). The coefficients of determination (R^2^) are indicated and significance *p* was <0.001 in all cases.

Spectral density of the first few MAF_(n)_ was composed mainly by low-frequency signals (e.g. ~32 and 19 years). The spectral regions appearing repeatedly where; 28–34, 17–20 and 9–11 years (Figs 6 and 9). According to the evidence and the theoretical context, those low-frequency signals might be associated to 33, 18.6 and 11 year periodicities observed in some large scale terrestrial and astronomical phenomena (Fig 9).

Based on spectral analysis results and the hierarchical arrangement of the MAFs (from high to low autocorrelations), first MAFs_(1…3)_ are representing better the longest periods (e.g. ~30, 25 and 19 years), while the rest MAFs_(4…6)_ kept higher frequencies (Fig 6).

Correlation coefficients in the first MAFs indicated a relative stronger influence of long periodicities at north (~45°N) and south (~24°N-21°N) bounds of the CC. In addition to signals of 33 and 19 years, the MAF-4, showed signals of ~16–11 years influencing upwelling/downwelling fluctuations in the CC and AC (Figs 5 and 6). The MAF-4 displayed the highest correlation coefficient of determination when fitting harmonics in annual resolution (R^2^ = 0.71) and the second highest (R^2^ = 0.64) when fitting from monthly values. The relative success of both temporal resolutions may be indicating the importance of detected frequency spectrum (~30–11 years; Fig 6), particularly at those latitudes with significant correlations (21°N, 30°N-42°N and 60°N; Fig 5).

Spectral density had an even distribution in time series at the domain of AC (57°N-60°N), where annual downwelling average is typically high (~ -500 m^3^/s/100_m coastline_). Fading of low-frequency signals in northern locations (captured in MAF-6), suggest two possible explanations: 1) the dissipation of low-frequency signals in a south-north gradient, or 2) the strength of interannual variability originated by the Aleutian Low Pressure Center (closer to AC), vanishes the sign of low-frequencies. The MAF-6 also provides evidence of connections operating at multiple time scales (e.g. ~ 11, 19, 25 years) between distant regions. Such regions characterize by having the highest values of yearly Ekman transport, but their directions are opposite: offshore (promoting upwelling) at 33°N-36°N ($\bar{x}$ = 1479 m^3^/s/100_m coastline_), and inshore (generating downwelling) around 57°N-60°N ($\bar{x}$ = -648 m^3^/s/100_m coastline_).

**Fig 10:**
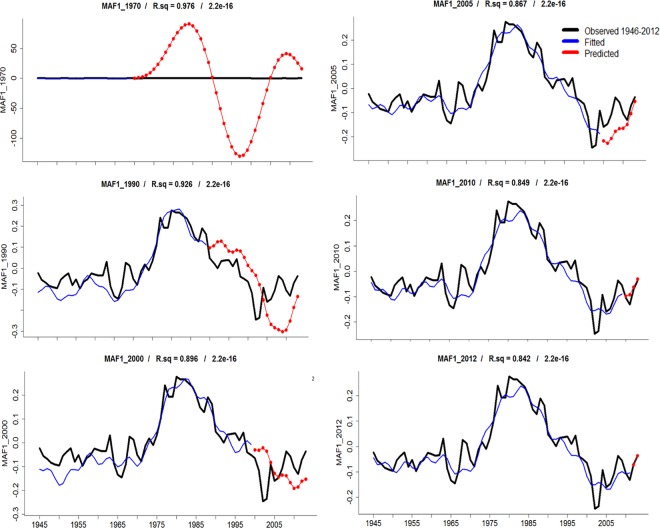
Assessment of the *MAF*-1 predictive skill using the most persistent (low-frequency) changing patterns in periodic regressions forecast. The black line shows the *MAF*-1 computed from observed values, the gradually increased-observations in hindcast is presented in blue, while the red line and dots show the predicted values.

**Fig 11:**
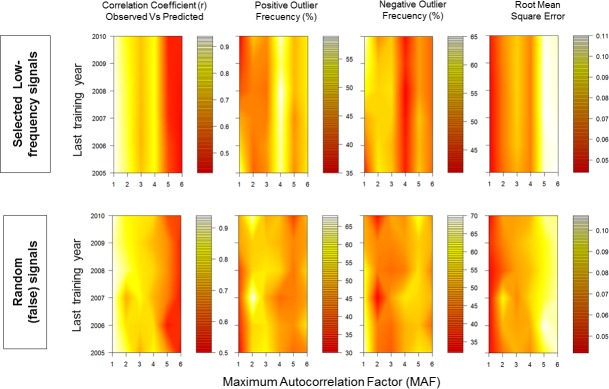
Skill assessment statistics from nowcast models (periodic regressions). Left column shows the performance of low frequency signals selected at present work versus (Right column) the performance of (false) periodicities taken from random numbers in the range 1:100, in the same quantity that selected signals (five).

The set of periodicities that we found (Fig 9) were consistent in nowcast/hindcast models, regardless of varying length of time series during model training (Figs 10 and 11). When projections were five years or longer, problems were observed in computations of the phase, amplitude and the direction of change (Fig 10); which did not happened in the analysis of skill statistics for all MAF(n) (Fig 11). Careful selection of low-frequency signals, in contrast to random values, yielded consistent results during skill assessment (as visualized in contour maps; Fig 11). Such consistency suggests the possibility to develop some forecasting skill using signals extracted from macro-scale upwelling/downwelling activity at North American west coast. In complement, the nowcast/hindcast assessment of MAF-1 suggests a modest skill about four years forward (in absence of El Niño conditions; Fig 10).

## 4. Discussion

### 4.1. Autocorrelation and long term change

Upwelling/downwelling variability along North American west coast over different time-scales impact the physical environment where many commercial marine populations develop their life stages [48]. Studies on long-term fluctuations have found long ranging trends related to environmental changes that affect recruitment, thus inducing fluctuations in stock size and biotic processes, such as predation, cannibalism and competition [49]. Long range dependence or long memory is a feature of many processes in the natural world, which provides important insights about underlying mechanisms [49,50].

Exploration of climatic processes presenting long range dependence can be enhanced using Min/Max Autocorrelation Factor Analysis (MAFA). MAFA is an advantageous technique (*e*.*g*. in comparison to Principal Components) because optimizing autocorrelation highlights the data structure in the domain of time (Fig 3). According to [51] in theoretical nonlinear systems (*e*.*g*. about ocean climate), resonances between intrinsic high-frequency fluctuations in small parts of a system and external forcing with additional energy supply will lead to low-frequency large-scale fluctuations through non-linear long-range interactions.

### 4.2. Spatial and temporal response

Low-frequency signals identified in upwelling/downwelling MAF(n) (Fig 9) are coherent with cycles previously reported in biological and physical variables at the study region: Sea surface temperature at the Pacific coast of North America [31], catch fluctuations of anchovy *Engraulis mordax* and sardine *Sardinops sagax* [52], the Pacific Decadal Oscillation [53], abundance and distribution changes of salmon populations [53,54] and variations in the position and intensity of the Aleutian low pressure center [53–55].

Fig 9 shows some similarities of the low-frequency signals that conform some portion of MAF(n) variability. Those MAF(n) contain information of spatial co-variability from original upwelling index time series but, at the same time, show latitudinal heterogeneous relationships (Figs 4 and 5). Therefore, each MAF might be understood as geographic expressions of common climatic signals. In other upwelling regions, at the north and center of Chilean coasts, [56] also described spatially heterogeneous expressions of Ekman Transport in response to climatic forcing.

[10] mention that low frequency component detected by singular spectral analysis, explained better upwelling variance at CC south of ~24°N, decreasing towards north. The south-north gradient coincides with our results because lowest-frequency signals (>20 years) represented in the first MAFs_(1…3)_, are stronger at those stations between 21°N and 33°N (Figs 4 and 6).

Decadal and bi-decadal frequencies were better captured by MAFs 4 to 6, with higher contributions from locations at the center (~36°N-42°N) and north (~57°N-60°N) of the study area; this patterns coincide with the explained variance in the south (87%) and north (50%) areas defined by [10]. The consistency of decadal and bi-decadal signals and its expression in south and north areas, might be related to the influence of the large-scale atmospheric pressure dynamics, which has been reported as the main forcing factor of interannual change at all North Pacific basin [23,57].

### 4.3. Large-scale forcing

Despite fluctuations of higher frequency (<10 years; Figs 4–6), large-scale and long-term forces are fundamental components of variations in direction and intensity of Ekman transport (Fig 3). For example, the synchronic and rapid change in upwelling activity captured by MAF-1 at mid-1970s resembles physical changes in the Pacific climate during the regime shift of years 1976–79 (Fig 7) [58], coinciding with changes in other upwelling areas around the planet [59–61]. The magnitude of Ekman´s transport is determined by winds force/direction and thermocline depth; both factors present macro-scale long-term patterns [55,62,63], suggesting potential low-frequency interactions with oceanic and atmospheric tides [64,65].

In the atmosphere, the response to tidal effects involves changes in the location and intensity of large pressure centers [63,66,67]. Long-term changes in the pressure gradient formed between the Aleutians low and the high of Hawaii promote major/minor friction of winds from the north, which favor/reduce the offshore component of Ekman transport along the west coast of North America [10,55,68]. This atmospheric process is related to the cold and warm phases of the North Pacific, which in turn contribute to the fluctuations of the low-pressure center in the region [69,70]. Meanwhile, tidal forces in the ocean implicate changes in water masses distribution that manifests in changes of heat content, mean sea level and thermocline depth [64,71].

Several North Pacific climate indices also evidence the macro-scale context of Ekman´s transport variability in the study region (Fig 8). Climate indices and upwelling index correlates preferentially to low frequencies [10]. The PDO and the Pacific Circulation index (PCIwinter) contain low-frequency signals as inherent components of their variability, showing notable impacts over the North Pacific climate as well as fish populations dynamics [53,72,73].

The PDO correlated (r = -0.44) with the low frequency signal identified by [10]. In complement, it was reported that geostrophic wind and PDO relationship show a north-south gradient with high r values (>0.52) to the north of 40°N [28]. The authors reported for MEI-wind correlations a fluctuating gradient behavior with high values (r>0.5) at 35°N, gradually decreasing to the north but increasing again close to ~39°N, and again decreasing consistently to the pole. The gradient for NPGO-wind relationship was south-north, with high values (r >0.5) south of 37°N [28]. The NPGO has been described as a mode of atmospheric forcing that modulates decadal changes in upwelling, and simultaneously explains fluctuations in long-term salinity, nutrients, and chlorophyll observed at the CC [70].

Our study supports the idea that upwelling/downwelling activity at North American west coast has a macro-scale component controlled by the Pacific climate [10]. Additionally, we tried to move forward investigating possible connections of this macro-scale patterns with other phenomena that fluctuate at similar temporal scales.

### 4.4. Upwelling/downwelling intensity and low-frequency change

The 11 year period detected in upwelling/downwelling MAFs (Fig 9) was among the most persistent signals. This period (11 years) is a well-known pattern of variability in sun activity, suggesting a relationship with upwelling: the sun drives thermal differences that modify pressure gradients and associated wind patterns [8,28,74,75]. The equatorial region acts as the primary terrestrial receiver of solar irradiance, which is redistributed to the rest of the planet by oceanic and atmospheric currents [76,77]. The radiant energy captured at the equator and its redistribution is part of the process that underlies multiple tropical-extratropical interactions [70,78,79]. An example of dissipation mechanisms of radiant energy from the equator, is the teleconnection between El Niño Southern Oscillation (ENSO) and those intensity variations in the Alaska and California Currents at North Pacific [80].

Another changing pattern that was persistently identified across MAFs was the period of 19 years (Table 1), clearly in proximity to the 18.6 periodicity of the moon´s nodal cycle [81]. During this time, the plane on which the moon orbits the earth gradually tilts, driving regional differences in the gravitational attraction of the moon over water and air masses [82]. Then, in addition to smaller temporal scale processes, the nodal cycle promotes a response in the tides, regional mean sea level, and thermocline depth [81,83,84]. Both parameters (sea level and thermocline depth) are determinants in the magnitude of Ekman transport [62,83] and they are also influenced by moon tidal effects, as demonstrated in upwelling predictive models [85].

Changes in the distribution of large-scale air masses imply that in a period of ~30 years, the meridional wind component strengthens, then the cycle is completed after another 30 years in which the system transits from meridional to zonal circulation (also called climatic epochs) [63]. While this mechanism was originally described for the Atlantic, the large-scale atmospheric circulation at the North Pacific follows the same pattern (~30 years), whereby the zonal and meridional wind components alternate [55,72]. This cycle is related to the cold and warm phases of the North Pacific, which in turn contribute to air pressure fluctuations in the region [67].

Changes in the pressure gradient between the Aleutians low and the north Pacific high induce major/minor friction of winds from the north, which favor/reduce the upwelling/downwelling intensity in the CC and AC [10,55,70]. The variations in the Earth’s rotational frequency could explain the link between upwelling and basin-scale climatic processes [64,67,81,86,87]. A meridional-zonal transition period lasts 60 years, which is the same duration of the dominant epoch of Earth´s rotation: acceleration intensifies zonal-dominated circulation at high atmosphere, but in response to friction between atmospheric layers, it favors the meridional wind component at the surface [63,67], thus stimulating Ekman´s transport. On the other hand, when Earth´s rotation rate decreases, the wind meridional component weakens at the surface (low atmosphere) and macro-scale upwelling/downwelling activity is depleted [6,67].

### 4.5. Summary

The recurring low-frequency signals (~30, 19, and 11 years) in the analyzed series may indicate a relationship (causal or not) with some natural events (Fig 9) as modulators of long-term/large-scale upwelling/downwelling activity. We hypothesize that upwelling/downwelling at North American west coast (and potentially at other eastern boundary currents), may be influenced on a regular basis by tidal effects (drag) originated by deviations of Earth’s rotational rate, via changes in the distribution of air and water masses [64,81,86]. Winds alternate meridional/zonal dominant components (named climatic epochs) with an approximate duration of 30 years [63,69], apparently driving a (spatially heterogeneous) multidecadal depletion/enhancement in upwelling/downwelling activity at Northamerican west coast. The lunar nodal cycle is observed regularly every ~18.6 years with effects on the position of pressure centers and, therefore, on regional wind patterns, as well as thermocline depth and sea level [62,88]. In addition, the solar cycle is ~11 years [89] and imprints its signature on sea-level pressure [77], suggesting a feasible link with the direction and intensity of Ekman´s transport, by the modulation of wind-stress. Finally, we found evidence that the vertical movements of sea water at the study region, share particular signals (*e*.*g*. 11 years period) as forcing patterns (Fig 9; Figs 6–8). Such fact suggests the possibility to extend in time those signals and build particular strategies to model upwelling/downwelling activity at the North American west coast. In this regard, a nowcast/hindcast approximation was exemplified at Fig 9 However, deepening a deterministic and theoretical framework to increase our understanding of macro-scale upwelling/downwelling activity, still remains to be consolidated.

## 5. Conclusions

- Recurring patterns of change (e.g. low-frequency signals) were observed across upwelling/downwelling MAFs, suggesting the presence of regional expressions of large-scale climatic forcing over Ekman´s transport direction and intensity at the west coast of North America.
- The relative importance of each detected low-frequency signal was variable, according to the geographic location, reflecting interactions of (underlying-common) large-scale to local climate conditions.
- The Ekman transport studied through the Upwelling index for the geographical domain 21–60°N contains low-frequency signals that vary in proximity to changes in long-term dominant winds, the lunar nodal cycle and solar irradiance. These natural phenomena are known to influence upwelling/downwelling at shorter time scales, for example: the predominance and intensity of zonal/meridional winds in the scale of days to weeks, and the lunar phase cycle that modulates water and air masses distribution in a daily and weekly basis (tides).
